# Sinus arrest and syncope in a young patient with Epstein–Barr virus infection: a case report

**DOI:** 10.1093/ehjcr/ytaf025

**Published:** 2025-01-23

**Authors:** Gianmarco Sarto, Beatrice Simeone, Marco Bernardi, Giacomo Frati, Sebastiano Sciarretta

**Affiliations:** Cardiology Division, ICOT Istituto ‘Marco Pasquali’ University Hospital, Via Franco Faggiana 1668, Latina 04100, Italy; Cardiology Division, ICOT Istituto ‘Marco Pasquali’ University Hospital, Via Franco Faggiana 1668, Latina 04100, Italy; Cardiology Division, ICOT Istituto ‘Marco Pasquali’ University Hospital, Via Franco Faggiana 1668, Latina 04100, Italy; IRCCS Neuromed, Via Atinense 18, Pozzilli 86077, Italy; Department of Medical-Surgical Sciences and Biotechnologies, Sapienza University of Rome, Corso della Repubblica 79, Latina 04100, Italy; IRCCS Neuromed, Via Atinense 18, Pozzilli 86077, Italy; Department of Medical-Surgical Sciences and Biotechnologies, Sapienza University of Rome, Corso della Repubblica 79, Latina 04100, Italy

**Keywords:** Epstein–Barr virus, Syncope, Sinus arrest, Conduction abnormalities, Pacemaker, Case report

## Abstract

**Background:**

Emerging evidence suggests a potential link between Epstein–Barr virus (EBV) infection and cardiovascular diseases. However, the impact of EBV infection on the development of arrhythmias and conduction abnormalities remains to be fully clarified.

**Case summary:**

We present a case report of a healthy 38-year-old Caucasian male who underwent an occupational medicine visit, during which a resting electrocardiogram (ECG) showed numerous premature ventricular contractions. He was later prescribed a stress test. During the test, he experienced a sinus arrest of 6.5 seconds with true syncope during the recovery phase. EBV infection was the only concomitant pathological finding observed during subsequent diagnostic investigations. Initially, it was necessary to rule out all possible cardiac causes of the event, especially in such a young patient. Comprehensive cardiac evaluations, including ECG, echocardiography, cardiac computed tomography, cardiac magnetic resonance, and electrophysiological studies, were normal. After 2 months, both the resting ECG and stress test were completely normal. The final diagnosis for the patient was ‘reflex cardioinhibitory syncope’. Accordingly, a pacemaker (PMK) device was not implanted, as the patient was under 40 years old and had no history of recurrent syncope, in accordance with European Guidelines.

**Discussion:**

Temporary conditions that may cause conduction abnormalities are a contraindication to PMK implantation. Therefore, it is crucial to always consider EBV infection in the differential diagnosis of cardiac conduction disorders. One hypothesis is that EBV may have specifically affected the sinoatrial node and a few right ventricular outflow tract cells without causing myocarditis signs.

**Learning points**

- Epstein–Barr virus (EBV) infection can result in arrhythmic abnormalities in the absence of signs of myocarditis.
- It is crucial to always consider EBV infection in the differential diagnosis of cardiac conduction disorders, in particular before considering pacemaker implantation.

## Introduction

Epstein–Barr virus (EBV), also known as human herpesvirus 4, is an enveloped, double-stranded DNA virus belonging to the gammaherpesviruses family. It is a pervasive human pathogen with a global prevalence exceeding 90% and primarily infects B lymphocytes, establishing a lifelong latent infection.^1^ EBV is associated with various clinical manifestations, ranging from infectious mononucleosis to more severe conditions, including different malignancies.^1^ However, emerging evidence also suggests a potential link between EBV infection and cardiovascular diseases.^2^ Cardiac injury related to EBV infection can manifest in several forms, such as coronary artery ectasia, coronary artery aneurysm, myocarditis, and heart failure.^3^ On the other hand, the impact of EBV infection on the development of arrhythmias and conduction abnormalities still needs to be fully clarified. Cardiac abnormalities associated with EBV infection may be transitory and, therefore, a rapid diagnosis is essential in order to avoid inappropriate medical interventions.

## Summary figure

**Figure:**
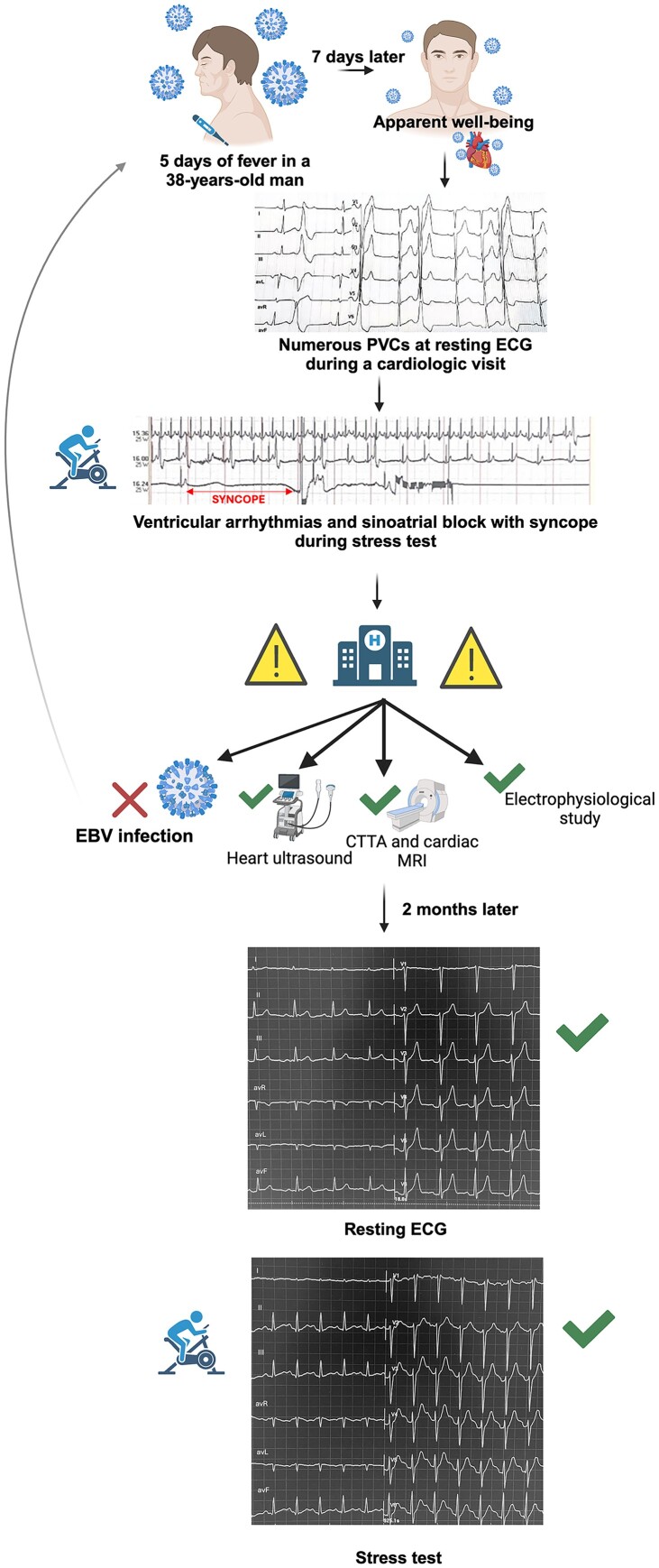


## Case presentation

**Figure 1:**
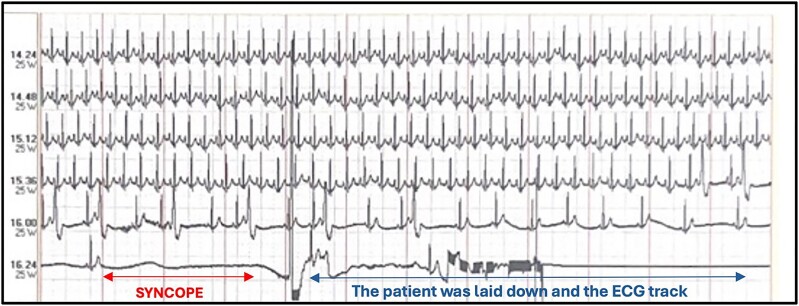
During the fourth minute of the recovery phase of the stress test, progressive bradycardia appeared with concomitant presence of numerous premature ventricular contractions, monomorphic, not early, which then resulted in complete sinoatrial block of 6.5 seconds with true syncope of the patient.

**Figure 2:**
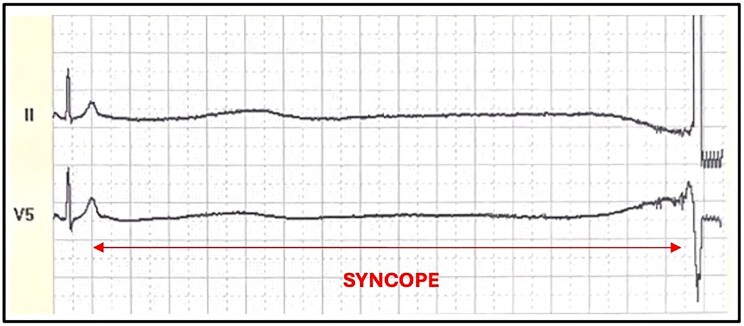
Complete sinus arrest for 6.5 seconds present also in *Figure 1*.

A 38-year-old Caucasian male with an unremarkable medical history underwent a routine occupational medicine examination, which revealed numerous premature ventricular contractions (PVCs) on a resting electrocardiogram (ECG). He was asymptomatic for angina, dyspnoea, and palpitations. Consequently, he was referred for a cardiological examination, where an ECG confirmed isolated PVCs. A transthoracic (TT) echocardiography showed no significant pathological findings. Subsequently, a 24-h Holter ECG showed a sinus rhythm (SR) throughout the recording, with frequent supraventricular ectopic beats (603) and very frequent isolated PVCs (13 000), monomorphic and not early. He underwent an exercise stress test at our Cardiology Unit, which revealed numerous monomorphic, non-early PVCs on the resting ECG, disappearing during exercise. During the fourth minute of the recovery phase, progressive bradycardia occurred, accompanied by numerous monomorphic, non-early PVCs, resulting in a complete sinoatrial block of 6.5 seconds with true syncope (*Figures 1* and *2*). The condition resolved spontaneously when the patient was placed in the supine position. The patient was promptly admitted to our Cardiology Unit for further investigations. On admission, his ECG showed SR at a heart rate (HR) of 95 beat per minute with numerous PVCs; TT echocardiography revealed an ejection fraction of 55% without clear segmental wall motion abnormalities. Physical examination was unremarkable. The family history was unremarkable, as was the remote medical history, with no prior episodes of syncope. Laboratory tests revealed leukocytosis with 15 600/μL white blood cells, with 76.9% lymphocytes. Liver function tests showed elevated AST [124 U/L (n.v. 17–59)], ALT [316 IU/L (n.v. 0–50)], GGT [519 IU/L (n.v. 15–73)], and ALP [282 U/L (n.v. 38–126)]. Bilirubin levels were within normal ranges. No signs of dehydration were detected, with normal blood urea nitrogen, haematocrit, electrolytes, and creatinine. On a thorough remote pathological history investigation, he was found to have had 5 days of fever, about 1 week before the admission, which was initially attributed to a simple flu syndrome. Specific tests for hepatotropic and related viruses, as well as an abdominal ultrasound, were performed. Hematochemical examinations showed positive IgG and IgM antibodies for EBV (IgG ANTI EBV-VCA 70 U/mL [n.v. < 20 U/mL]; IgM ANTI EBV-VCA >160 U/mL [n.v. < 40 U/mL]), while the abdominal ultrasound and computed tomography (CT) scan of the thorax and abdomen showed hepatosplenomegaly and lymphadenomegaly. The high sensitive troponine value was <1.5 ng/L [n.v. 1.50–11 ng/L], the creatine kinase myocardial band (CK-MB) 5 U/L, creatine phosphokinase (CPK) 46 U/L [n.v 55–170 U/L], the NT-proBNP was 80 pg/mL. Electrolytes were within normal limits. Coronary CT (CCT) and cardiac magnetic resonance (CMR) were conducted to exclude any cardiologic pathological substrate explaining the complete heart block; both were normal. A new color Doppler TT echocardiogram during the hospital stay was consistent with the admission findings. Considering the patient's young age and the first syncopal episode with a long pause on the ECG, an electrophysiological study was conducted, showing no significant sinus atrial or atrioventricular conduction defects, with a normal sinus node recovery time. A loop recorder was implanted. On the other hand, carotid sinus massage and tilt tests were not performed since a diagnosis of reflex cardioinhibitory syncope was already made, with a recorded sinus arrest of more than 6 seconds, and they would have represented potentially risky and stressful procedures for our patient, without a clear advantage in terms of clinical management.

The patient was discharged asymptomatic and haemodynamically stable, with instructions to rest for at least 2 months until re-evaluation with a stress test at our outpatient clinic. Two months later, the patient underwent the stress test, achieving 99% of his HR, remaining asymptomatic with no PVCs or pauses. To date, 13 months after discharge, the loop recorder ECG showed no relevant conduction disturbances or arrhythmias.

## Discussion

We present a case report of a 38-year-old Caucasian male with an unremarkable medical history who experienced a sinus arrest of 6.5 seconds with true syncope during the recovery phase of a stress test. Recent EBV infection was a concomitant pathological finding observed during subsequent diagnostic investigations in our patient. This clinical case offers several points for reflection.

The final diagnosis we posed was ‘reflex cardioinhibitory syncope’, since the episode was associated with a sinus arrest of more than 6 seconds and occurred after a strenuous physical exertion.^4^ Since the syncope did not occur at rest and the patient did not have a history of rest syncope or bradyarrhythmia, we did not consider a diagnosis of sinus node dysfunction (SND).

In such a young subject with very frequent PVCs and prolonged sinus arrest, we had to confidently exclude the presence of heart disease, and, therefore, in addition to ECG and echocardiography, we also performed CCT, CMR, and an electrophysiological study, which resulted normal. We did not consider the option of implanting a pacemaker device because the patient had no history of recurrent syncope and was <40 years of age, as indicated by the European guidelines.^5^ We might have considered a pacemaker device implantation if the subject was >40 years old with multiple episodes of recurrent reflex cardioinhibitory syncope.^5^ In addition, in case of SND, the European guidelines recommend considering pacing when syncope is caused by bradyarrhythmias, if no temporary conditions are identified.^5^ Indeed, it is always important to exclude transient causes of conduction disease that can be corrected or prevented, as pacing is not recommended in such cases.^5^

We believe that acute EBV infection was involved in the development of the reflex cardioinhibitory syncope and frequent resting PVCs in our patient, given the fact that 2 months later, after the complete resolution of the viral infection, the ECG trace both at rest and during the stress test was completely normal. In addition, the resting PVCs of our patient had an inferior axis with a left bundle branch block morphology, typical of PVCs from the right ventricle outflow tract (RVOT). In the literature there is a correlation between the embryogenesis of cells that give rise to RVOT PVCs and sinus atrial node (SAN) cells.^6^

A similar embryogenetic origin could explain a specific tropism of the virus for this cell type. The numerous PVCs could be explained by the viral irritation of these cells in the RVOT, and the long sinus arrest could be justified by a subtle dysfunction of the SAN cells, which was precipitated by a strenuous physical exercise and a vasovagal trigger. Future studies are warranted to test this hypothesis.

Previous work reported a correlation between EBV and myocarditis.^7^ Arrhythmias are well-known consequences of myocarditis, ranging from ventricular arrhythmias to atrial fibrillation, atrioventricular blocks, and complete heart blocks.^8^ However, our patient did not show signs of overt myocarditis, since both C-reactive protein and troponin were normal, and the CMR showed no significant findings. Therefore, it is possible that in our patient EBV was limited to specific myocardial areas, such SAN and RVOT, sparing the rest of the myocardial tissue. Another possibility is that myocardial EBV infection was at the end of the acute phase, which could explain the normal troponin level and the absence of oedema on CMR. In fact, CMR has high sensitivity for detecting oedema if performed within 2 weeks of acute infection.^8^ Interestingly, there are reports in the literature associating acute viral infections, including EBV, with a transient derangement of autonomic function, which may have also played a role in the development of reflex syncope in our patient.^9^ The presence of resting premature ventricular beats that disappear during exercise may also support this possibility in our case. On the other hand, temporary electrolyte imbalances secondary to viral infection could be excluded in our patient since sodium, potassium, and chlorine values were in the normal range.

In the literature, several cases describe a correlation between EBV infection and arrhythmic disorders, primarily focusing on ventricular arrhythmia and cardiac arrest.^10,11^ However, in all reported cases, myocarditis was present, as evidenced by both laboratory findings and imaging. Of note, only one case in the literature documented a correlation between complete heart block and acute EBV infection, in the absence of myocarditis, but in the presence of pericarditis.^12^ To our knowledge, our case represents the first description in the literature of a reflex cardioinhibitory syncope in a patient without overt heart disease, including myocarditis.

## Conclusion

In conclusion, we think that it is crucial to consider the presence of acute EBV infection in patients with syncope associated with bradyarrhythmias, before considering a pacemaker implantation. Future studies are needed to better understand the pathogenic mechanism between EBV infection and heart conduction diseases.

## Data Availability

The data underlying this article will be shared on reasonable request to the corresponding author.

## Lead author biography

Dr Gianmarco Sarto is a Cardiology Fellow at Sapienza University of Rome, currently working at Cardiology Division, ICOT University Hospital in Latina. He completed his basic medical degree in 2021 from Sapienza University of Rome with the highest grades. Immediately after graduation he won a fellowship to perform a traineeship at the Cardiovascular Division of the University of Oxford, before starting the current fellowship program.

**Consent:** The authors confirm that written consent for submission and publication of this case report, including the accompanying images and associated text, has been obtained from the patient in line with the COPE guidelines.

**Funding:** There were no external sources of funding.
